# Direct Versus Indirect Causation as a Semantic Linguistic Universal: Using a Computational Model of English, Hebrew, Hindi, Japanese, and K'iche’ Mayan to Predict Grammaticality Judgments in Balinese

**DOI:** 10.1111/cogs.12974

**Published:** 2021-04-20

**Authors:** I Nyoman Aryawibawa, Yana Qomariana, Ketut Artawa, Ben Ambridge

**Affiliations:** ^1^ Udayana University; ^2^ Institute of Population Health Sciences University of Liverpool

**Keywords:** Syntax, Morphology, Balinese, Causativity, Causation, Computational modeling

## Abstract

The aim of this study was to test the claim that languages universally employ morphosyntactic marking to differentiate events of more‐ versus less‐direct causation, preferring to mark them with less‐ and more‐ overt marking, respectively (e.g., *Somebody broke the window* vs. Somebody MADE the window break; *Somebody cried the boy vs. *Somebody MADE the boy cry*). To this end, we investigated whether a recent computational model which learns to predict speakers’ by‐verb relative preference for the two causatives in English, Hebrew, Hindi, Japanese, and K'iche’ Mayan is able to generalize to a sixth language on which it has never been trained: Balinese. Judgments of the relative acceptability of the less‐ and more‐transparent causative forms of 60 verbs were collected from 48 native‐speaking Balinese adults. The composite crosslinguistic computational model was able to predict these judgments, not only for verbs that it had seen, but also––in a split‐half validation test––to verbs that it had never seen in any language. A “random‐semantics” model showed only a relatively small decrement in performance with seen verbs, whose behavior can be learned on a verb‐by‐verb basis, but achieved zero correlation with human judgments when generalizing to unseen verbs. Together, these findings suggest that Balinese conceptualizes directness of causation in a similar way to these unrelated languages, and therefore constitute support for the view that the distinction between more‐ versus less‐distinct causation constitutes a morphosyntactic universal.

Human languages allow their speakers to express any concept they can conceive of. But some concepts are apparently so ubiquitous in human experience that languages afford them dedicated morphosyntactic constructions. Consider the example of causation, which boasts a dedicated construction in most––perhaps even all––languages (e.g., Comrie, 1976; Comrie & Polinsky, 1993; Dixon, 2000; Haspelmath, 1993; Nedjalkov, 1969). English speakers do not need to use an awkward circumlocution like *André hit the window, which caused it to break*. Instead, they can use their language's *transitive causative* construction to say *André broke the window*. So ubiquitous, in fact, is human discussion of causation that many languages––Shibatani and Pardeshi (2002) list 38 examples^1.^––offer their speakers not one but two dedicated causative constructions, which express different degrees of causation.

For example, in addition to the *transitive causative* construction, English has the *periphrastic causative* construction (e.g., *André made the window break*). As its name implies, this construction denotes a more indirect, roundabout kind of causation than the *transitive causative*. For example, if André deliberately hit the window with a hammer, it would be much more natural to say *André broke the window* than *André made the window break*. But if André startled the window fitter, causing him to drop the glass, it would be much more natural to say *André made the window break* than *André broke the window*. Indeed, some verbs/events require a direct cause to such an extent that they more‐or‐less prohibit the less‐direct causative (e.g., Someone stole the jewellery; *Someone made the jewellery steal). Others are so resistant to direct causation that they more‐or‐less prohibit the more‐direct causative (e.g., Someone made the boy cry; *Someone cried the boy).

One of the most comprehensive typological summaries of the way that languages differentiate more‐ and less‐direct causative types is provided by Dixon (2000), as summarized in Table 1 (see also Comrie, 1981; Song, 1996; Talmy, 2000).

**Table 1:** More‐ and less‐direct causative types across languages

	Meaning		Mechanism		
Parameter	Causative Type 1	Causative Type 2	Causative Type 1	Causative Type 2	Language
1	State	Action	Morphological_1_	Morphological_2_	Amharic
			Morphological	Periphrastic	Bahasa Indonesian, Malay
2	Intransitive	All transitive	Morphological	Periphrastic	Austronesian languages, Mayan languages, etc.
	Intransitive and simple transitive	Ditransitive	Morphological	Periphrastic	Basque, Abkhaz
3	Causee lacking control	Causee having control	Lexical	Morphological	Japanese
			Morphological_1_	Morphological_2_	Creek
4	Causee willing	Causee unwilling	Morphological_1_	Morphological_2_	Swahili
			Morphological	CPeriphrastic	Tangkhul Naga
			Morphological	Periphrastic	Swahili
5	Causee partially affected	Causee fully affected	Morphological_1_	Morphological_2_	Tariana
6	Direct	Indirect	Morphological_1_	Morphological_2_	Nivkh, Apalaí, Hindi, Jingpaw
			Morphological	Periphrastic	Buru, Chrau, Alamblak, Mixtec, Korean
7	Intentional	Accidental	Morphological	CPeriphrastic	Kammu
			Periphrastic	Morphological plus periphrastic	Chrau
8	Naturally	With effort	Lexical	Morphological	Fijian
			Lexical	Periphrastic	English
			Morphological	Periphrastic	Russian, Tariana

Note: 1 and 2 refer to more‐ and less‐ compact morphological processes respectively (terms from Dixon, 2000).

Although there has been a great deal of debate as to how best to characterize the two different causative types (we summarize them simply as “more direct” and “less direct,” corresponding to Dixon's Type 1 and Type 2, respectively), the claim that languages universally mark such a distinction morphosyntactically enjoys considerable typological support. However, to our knowledge, no study (except, in part, the study of Ambridge et al., 2020, summarized below) has tested this claim experimentally.

The aim of the present study is, therefore, to test a version of this claim; specifically, the claim that actions that are amenable to more‐ and less‐direct causation tend, across languages, to strongly prefer what Shibatani and Pardeshi (2002) term *less‐transparent* and *more‐transparent* causative forms, respectively. We do so by investigating whether a recent computational model which learns to predict speakers’ by‐verb relative preference for the two causatives in English, Hebrew, Hindi, Japanese, and K'iche’ Mayan (Ambridge et al., 2020) can generalize to a sixth language, on which it has never been trained: Balinese.

Ambridge et al. (2020) tested the particular formulation of this *semantic‐universal* hypothesis set out by Shibatani and Pardeshi (2002: 89), though the general idea dates back at least as far as Fodor (1970), Givon (1979) and Hopper and Thompson (1980):

- Less‐direct causation entails an event in which “both the causing and the caused event enjoy some degree of autonomy…The caused event … may have its own spatial and temporal profiles distinct from those of the causing event,” and hence is associated with *more‐trans‐ parent* causative marking (e.g., the English *periphrastic causative* construction, in which some form of the verb MAKE serves as a transparent causative marker, similar, for example, to Hindi *‐aa* or Japanese *–(s)ase*).
- More‐direct causation “entails a spatio‐temporal overlap of the causer's activity and the caused event, to the extent that the two relevant events are not clearly distinguishable,” and hence is associated with ***l*** *ess‐transparent* causative marking (e.g., the English *transitive causative* construction, which lacks a transparent causative marker, such as MAKE, similar, for example, to so‐called lexical‐causatives in Hindi or Japanese).

Ambridge et al. asked 20 native adult speakers of each language to rate 60 causative events—shown as unlabeled animations––for the extent to which they exhibit this property of *Event‐Merge*, as well as three further relevant properties set out by Shibatani and Pardeshi (2002): (a) *Autonomy* of the causee, (b) whether the caused event *Requires* a causer, and (c) whether causation is *Directive* (e.g., giving an order) or physical. The authors then trained a distributional‐learning computational model to predict the most probable of two causative forms for each of 60 corresponding verbs, presented in proportion to their frequency in suitable corpora for each language (noncausative forms in the corpus were mapped to an “Other” construction). Verbs were presented to the model as an orthogonal “lexical” vector, alongside their semantic properties as determined by the human semantic rating task. The model was then tested on its ability to predict participants’ relative preference for the more‐ versus less‐transparent causative construction for each verb, as obtained using a second judgment task with new adult participants. Even though the model was never presented with any grammaticality judgment data, it successfully learned to predict participants’ judgments with correlations of around *r* = .75. Importantly, the semantic information learned by the model allowed it to show similar performance on a split‐half validation task consisting of verbs on which it had never been trained.

Ambridge et al. (2020) built separate computational models for each language. However, if it is indeed the case that languages are highly similar in the way that they carve up semantic space into events of more‐direct and less‐direct causation, a composite model trained using semantic and frequency data from English, Hindi, Hebrew, Japanese, and K'iche’ Mayan should be able to make a reasonable stab at predicting human grammaticality judgments from a different language altogether. Here, we test this claim using Balinese.

Although an important motivation for choosing Balinese was of course the availability of native‐speaking participants and researchers, Balinese is highly suitable for our purposes given that, as an Austronesian language, it belongs to a different family to all five of the languages used to train the model. Thus, we can be confident that the predicted effect, if found, reflects the fact that Balinese has independently developed more‐ and less‐direct causative constructions along the continuum posited, rather than having inherited these constructions from one or more of our training languages.

Despite having a relatively small number of native speakers, Balinese is well documented in terms of the relevant morphosyntactic properties (e.g., Shibatani, Artawa, Malchukov, & Comrie, 2015). Similar to English, more‐transparent “indirect causation…calls for a periphrastic construction with the causative verb derived from the verb *ngranang* ‘make’” (p. 926)

(1) Anak ento ngranang anak‐e cenik ngeling

Someone that made person‐DEF small cry

“Someone made the child cry”

Again, similar to English, verbs denoting actions that are amenable only to relatively indirect causation (e.g., *ngeling*, “cry”) require this *periphrastic causative* and resist the standard *transitive causative* (which, in Balinese, requires the causative marker *‐ang*)

(2) *Anak ento ngeling‐ang anak‐e cenik*

*Person that cry‐CAUSE person‐DEF small

“*Someone cried the child”

Thus, the goal of the present study was to investigate whether a computational model trained on causatives in English, Hebrew, Hindi, Japanese, and K'iche’ Mayan could predict Balinese speakers’ relative preference for periphrastic versus transitive causatives across 60 verbs.

## 1 Method

### 1.1 Computational model

The computational model used to generate predictions of Balinese speakers’ judgments is a neural network model. However, unlike most modern neural network models, it has no hidden layers. Rather, it is a perceptron‐style discriminative learner (e.g., Widrow & Hoff, 1960). When stimuli are presented in random order, as in the present case, such models converge at something very close to the maximum likelihood estimate, equivalent to a simple regression model (e.g., Milin et al., submitted).^2.^ The reason we favor a discriminative‐learning architecture is that such models are well grounded in human and animal learning generally (e.g., Gureckis & Love, 2010; Rescorla, 1998; Rescorla & Wagner, 1972), and so enjoy psychological plausibility as models of learning. That is, such models constitute an approximation of how a neural system might actually accomplish regression. Discriminative learning models also offer a close fit to human data in a number of linguistic domains, including grammatical gender (Arnon & Ramscar, 2012), word‐learning (e.g., Baayen, Chuang, Shafaei‐Bajestan, & Blevins, 2019; Milin, Divjak, & Baayen, 2017; Ramscar, Dye, & Klein, 2013a), reading (e.g., Milin et al., 2017), and both inflectional and derivational morphology (e.g., Ramscar & Yarlett, 2007; Ramscar et al., 2013; Milin, Divjak, Dimitrijević, & Baayen, 2016; Durdevic & Milin 2019; Baayen & Smolka, 2019).

In terms of architecture and parameters, the present model was identical to each of the language‐specific models reported in Ambridge et al. (2020), from which the following description is taken:

The input to the model is a vector of 60 lexical units (1/0), a causative unit (1/0) and four semantic units (continuous activation level 0–1). The orthogonal lexical units represent the identity of the verb, and can be conceptualized as a pseudo‐phonological representation (e.g. the root /bɔɪl/ for English *boil*) and/or a pseudo‐lexical‐semantic representation (e.g., “[of a liquid] to heat until it reaches boiling point”). The causative unit (1/0) indicates whether or not the utterance presented to the model on that trial conveys causation. That is, the causative unit is set to 1 if the corpus utterance uses either the more‐ or less‐transparent causative form for the relevant verb (e.g., *The man made the water boil; The man boiled the water*) and to 0 if it does not (e.g., *The water boiled*). This unit can be conceptualized as representing, at a very broad‐brush level, event‐level semantics. The four semantic units, Event‐Merge, Autonomy, Directive and Requires, are assigned a continuous activation level based on the mean rating…for the verb in the relevant utterance. These units can be conceptualized as representing some subset of the overall semantics (i.e., verb‐level and/or event‐level semantics) of the relevant utterance. Finally, the orthogonal output units (with softmax activation function) are each set to 1 or 0 representing the form/utterance type of the relevant corpus utterance: More transparent (e.g., *The man made the water boil*), Less transparent (*The man boiled the water*) or Other (e.g., *The water boiled*).

The composite model built here differs from the language‐specific models reported in Ambridge et al. (2020) in two respects. First, the relative frequency with which each “utterance” was presented to the model was determined not on the basis of not a language‐specific corpus, but of a composite corpus created by randomly sampling 300,000 utterances from each of the English, Hebrew, Hindi, Japanese, and K'iche’ corpora (although, as in Ambridge et al., 2020, only 10,000 randomly sampled utterances were used for each model run). Second, and similarly, the continuous activation levels of the semantic units for each verb were determined not on a language‐specific basis, but by taking the mean rating across all 100 participants who completed the semantic rating task; 20 per language.

### 1.2 Ethics

The study was approved by the ethics committee of Udayana University, Bali, Indonesia.

All participants gave informed consent.

### 1.3 Participants

Forty‐eight native Balinese speaking adults took part in the grammaticality judgment task. Although formal language data were not collected, like all Balinese speakers, these participants would have also been fluent speakers of Indonesian, and most would have had basic level knowledge of English.

### 1.4 Materials and procedure

Participants rated the grammatical acceptability of 120 sentences presented by the experimenter in random order, using PsychoPy2 (Peirce et al., 2019): Each sentence included a more‐transparent (*ngranang*, “make” periphrastic) or less‐transparent (transitive) causative form of one of the same 60 verbs (in translation) used in Ambridge et al. (2020), with *Anak*, “someone” as the subject (e.g., 1–2). Each sentence was accompanied by an animation in which the caused event, but not the causer, was visible; the same animations used by Ambridge et al. (2020) for all languages, for both the grammaticality judgment and semantic rating tasks (downloaded from https://osf.io/pavm7/). Participants rated the acceptability of each sentence using a computerized 5‐point smiley‐face Likert‐type scale (designed originally for use with children).

## 2 Results

**Fig. 1:**
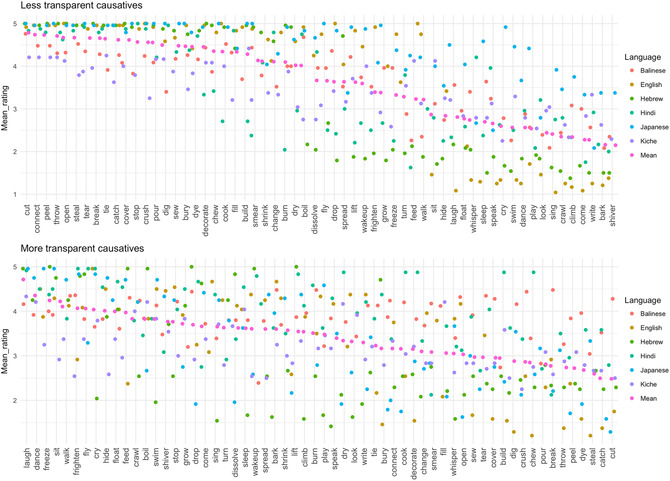
Participants’ mean ratings for less‐ and more‐transparent causative forms of 60 verbs (in translation equivalent) in Balinese (present study), English, Hebrew, Hindi, Japanese, and K'iche’ (data from Ambridge et al., 2020).

Mean ratings of the acceptability of the less‐transparent (transitive) and more‐transparent (periphrastic) causative forms of each verb are shown in Fig. 1, along with the corresponding adult data from English, Hindi, Hebrew, Japanese, and K'iche’ Mayan (note that these figures did not appear in Ambridge et al., 2020, but have been created using the raw data available at https://osf.io/pavm7/; the OSF repository for Ambridge et al., 2020). The code used to generate Fig. 1 can be downloaded from the OSF repository for the present study (https://osf.io/5sd2h/); specifically the file Balinese Fig 1. R from within the zip file Balinese_Modeling.zip. Visual inspection of Fig. 1 suggests that, as predicted by the semantic‐universal hypothesis, Balinese is generally similar to these other languages with regard to the acceptability of particular verbs in less‐ and more‐transparent causative forms. For example, across languages, *cut* tends to be highly acceptable in the less‐transparent causative form (top panel), and Balinese is no exception. Similarly, across languages, *laugh* tends to be highly acceptable in the more‐transparent causative form (bottom panel), and again Balinese is no exception. That said, Balinese does seem to differ from the other languages in that more‐transparent causatives receive relatively high acceptability ratings across the board. Indeed, of the 144 individual more‐transparent causative forms that receive acceptability ratings of less than 3.0 (the midpoint of the scale), only three (*build, pour*, and *wake up*) are Balinese. It is impossible to know whether this reflects a genuine property of Balinese, or simply a tendency toward leniency on the part of Balinese participants (who generally avoided the very lowest ratings across the board, unlike––in particular––English speakers).

In order to test this prediction in more detail, we investigated whether our computational model trained on causatives in English, Hebrew, Hindi, Japanese, and K'iche’ Mayan could predict Balinese speakers’ relative preference for the two forms of each verb. We did so by conducting simple model‐human Pearson calculations for each epoch of the model, in each case taking the mean rating for each verb across all 48 Balinese‐speaking participants and 48 runs of the model. The model code can be downloaded from the OSF repository for the present study (https://osf.io/5sd2h/); specifically the file V1 Balinese Model Original.R from within the zip file Balinese_Modeling.zip.

**Fig. 2:**
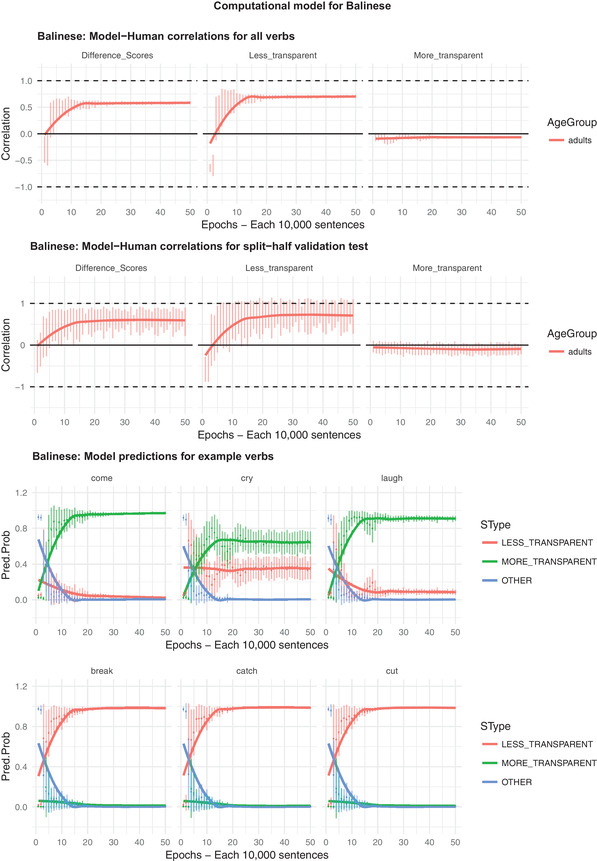
Correlation between predictions of composite crosslinguistic model's and Balinese speakers’ acceptability judgments for (a) difference scores (preference for less‐ over more‐transparent causative form), and raw ratings for (b) less‐transparent (transitive causative) and (c) more transparent (periphrastic causative) forms. Error bars show one standard deviation.

The results of this analysis are shown in Fig. 2 for (a) difference scores (preference for less‐ over more‐transparent causative form), and raw ratings for (b) less‐transparent (transitive causative) and (c) more transparent (periphrastic causative) forms. The X axis shows the developmental “age” of the model in epochs (1–50). At the end of each epoch––that is, after every batch of 10,000 input sentences––the model is interrogated for its “prediction” of the acceptability of the more‐ and less‐transparent form of each verb (as well as the “other” form, although this is not shown in the main plots). That is, at the end of each epoch, the model––with learning switched off––was presented with each verb (i.e., the relevant combination of lexical units and verb semantic units) with the Causative unit set to 1, and the resulting activation levels of the More‐transparent, Less‐Transparent, and Other output units taken as its acceptability judgment for the relevant sentence (e.g., *Someone made the water boil; Someone boiled the water; The water boiled*). We then calculated a difference score by subtracting, for each verb, the model's activation level for the More‐transparent unit from its activation level for the less transparent unit. Finally, these activation levels were correlated with human judgments.

For the top half of Fig. 2, the Y axis shows the resulting Pearson correlations (error bars show one standard deviation), and how they change over development. These correlations are shown, first, for “seen” verbs––that is, for verbs that are presented to the model in both the training and test phases––and for “unseen” verbs: For each model run, 30/60 verbs––selected at random––form the training set, and the remaining 30, the test set. The second row of plots (labeled “split‐half validation test”) shows the model's performance on these unseen verbs only.

For the bottom half of Fig. 2, the Y axis shows––for six example verbs, chosen to typify verbs that generally prefer, respectively, more‐ and less‐transparent causative forms across languages––the raw activation level of the relevant output unit (Less Transparent / Raw Transparent / Other). This part of the figure shows how the crosslinguistic model's preference for more‐ versus less‐transparent causative forms of individual verbs changes across development, simulating both children's overgeneralization errors with less‐transparent causative forms, and the fact that such errors are virtually absent for more‐transparent causative forms (e.g., Bowerman, 1988; Ambridge & Ambridge, 2020).

Looking at difference scores, which Ambridge et al. (2020) argued constitutes “in many ways the fairest test of the model, which…makes relative rather than absolute predictions regarding the acceptability of the more‐ and less‐transparent causative forms,” the model achieves a respectable, medium‐sized correlation of around .5 (by around 15 epochs). This is short of the within‐language calculations reported by Ambridge et al. (mainly around *r* = .75), but it is still comfortably statistically significant, even at the more stringent *p*<.01 cutoff (Critical *r* (df = 58) value for *p*<.05 = 0.21; for *p*<.01 = 0.30 (one‐tailed test).

Notably, however, the model's success is driven exclusively by its performance when predicting participants’ ratings for less‐transparent causative forms (*r* = .73). For more‐transparent causative forms, its correlation with participants’ ratings remains at around zero (and, indeed, is never positive). The reason that the model performs so poorly for more‐transparent causative forms is that, as discussed with regard to Fig. 1, Balinese speakers––unlike speakers of the other languages under investigation––tend to rate such forms as highly acceptable across the board. It is unsurprising, then, that semantic and distributional factors that are predictive of the relative acceptability of more‐transparent causative verb forms in other languages are not so for Balinese. What the present findings cannot tell us, however, is whether Balinese is genuinely more productive than these other languages with regard to the morphosyntactic operations that generate and/or process more‐transparent causative forms, or whether Balinese speakers in general––or our participants in particular––are simply relatively tolerant of grammatically “unacceptable” forms.

Returning to difference scores, the model's impressive correlation with human judgments is maintained in a split‐half validation test in which its task is to predict acceptability judgments for verbs it has never seen (in any language) during training (30/60 verbs, selected at random for each of the 48 model runs at each epoch). That is, the previous analysis demonstrates that a model trained on the English, Hindi, Hebrew, Japanese, and K'iche’ equivalents of (for example) *come* and *break* can predict the relative acceptability of the Balinese periphrastic‐ and transitive‐causative forms of *come* and *break*. The split‐half validation test demonstrates that a model trained on the English, Hindi, Hebrew, Japanese, and K'iche’ equivalents of (for example) *come* and *break* can predict the relative acceptability of the Balinese periphrastic‐ and transitive‐causative forms of (for example) *cry* and *cut*. We additionally ran a four‐fold validation task in which a randomly selected fold (15 verbs) constituted the held‐out items. The results of this analysis are not shown here since they are extremely similar to those of the split‐half analysis (though the model's performance increases slightly as compared with the split‐half test––*r* = .6 with participants’ difference scores––reflecting the fact that it must make predictions for only 15, as opposed to 30, unseen verbs in any one run). The relevant plots can be downloaded from the OSF repository for the present study (https://osf.io/5sd2h/); specifically, the file BAL_Model 4 Folds.pdf from within the zip file Balinese_Modeling.zip (created using the R code V2 Balinese Original but 4 Folds.r, from within the same zip file).

In order to explore how the model achieves its performance, and in particular, how it generalizes to novel verbs, we ran a new “random‐semantics” version of the model. In this version, the semantic feature vector (comprising four features) is permuted across the identity of the verb (as defined by the one‐hot lexical vector). Thus, as in the original simulations, each verb has a consistent semantic profile across learning, and the structure of the original dataset is maintained with respect to the relationships between the four semantic features. The difference is that particular patterns of semantic features––the four human‐derived measures of directness of causality––are no longer predictive of the relevant frequency of more‐ and less‐transparent forms in the composite corpus. Thus, we would expect the random‐semantics model to show some degradation of performance with seen verbs, but still achieve a respectable correlation with human judgments, since it can still perform lexical learning. That is, it can still learn which verbs––as defined by the 60 orthogonal one‐hot “lexical” units, occur mainly with more‐ versus less‐transparent causative forms (or vice versa). However, the random‐semantics model would be expect to break down completely in the split‐half validation task, because it no longer has the ability to make appropriate predictions for unseen verbs on the basis of their semantics.

**Fig. 3:**
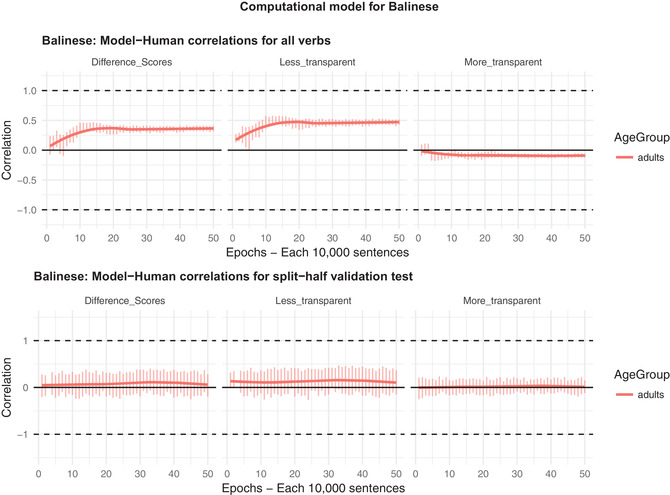
Correlation between predictions of the “no‐semantics” model's and Balinese speakers’ acceptability judgments for (a) difference scores (preference for less‐ over more‐transparent causative form), and raw ratings for (b) less‐transparent (transitive causative) and (c) more transparent (periphrastic causative) forms. Error bars show one standard deviation.

The results of the random‐semantics model are shown in Fig. 3 (for code, see the file V2 Balinese Random Semantics.R from Balinese_Modeling.zip at https://osf.io/5sd2h/). As expected, for seen verbs (top), the model still shows a reasonable, if reduced, correlation with participants’ judgments for difference scores (*r* = .33) and judgments for less‐transparent forms (*r* = .49), though, again, not more‐transparent forms (*r*<0). For unseen verbs, in the split‐half validation test, the model fails entirely, its correlation with predict participants’ judgments hovering around zero (well short of the critical value of *r* = .21 for *p*<.05). This pattern of findings suggests that a purely lexical learner can do a reasonable, if suboptimal, job of learning the extent to which particular verbs prefer which causative type. However, a purely lexical learner, almost by definition, has no basis on which to generalize to unseen verbs. This ability requires the introduction of semantic (or other; e.g., phonological) features that are predictive, to a lesser or greater degree, of the occurrence of each causative type (and, via the “Other” output unit, of none).

## 3 Discussion

A well‐supported claim in the typology literature holds that languages universally employ morphosyntactic marking to differentiate––at a broad‐brush level––events of more‐ versus less‐direct causation, preferring to mark them with less‐ and more‐overt marking, respectively. However, to our knowledge, no previous study has tested this claim experimentally. In the present study, we did so by investigating whether a recent computational model which learns to predict speakers’ by‐verb relative preference for the two causatives in English, Hebrew, Hindi, Japanese, and K'iche’ Mayan is able to generalize to a sixth language on which it has never been trained: Balinese.

In fact, this composite English…K'iche’ model was able to predict the relative acceptability not only of translational equivalent verbs in Balinese, but even––in a split‐half validation test––to verbs that it had never seen in any language. Comparison between the original and no‐semantics models demonstrates that the original model successfully generalized to unseen verbs on the basis of their semantics. That is, the model was learning which semantic properties predict corpus occurrences of more‐ versus less‐transparent causative marking universally (or, at least, across five typologically unrelated languages), and using them to predict the acceptability of causative forms in an untrained language. This pattern of findings, therefore, constitutes evidence in support of the semantic‐universal hypothesis.

## Funding

The author(s) disclosed receipt of the following financial support for the research, authorship, and/or publication of this article: This project has received funding from the European Research Council (ERC) under the European Union's Horizon 2020 research and innovation programme (Grant Agreement No. 681296: CLASS). Ben Ambridge is a Professor in the International Centre for Language and Communicative Development (LuCiD) at The University of Liverpool. The support of the Economic and Social Research Council [ES/L008955/1] is gratefully acknowledged.

## Notes

1. Ahtna, Alamblak, Amharic, Asheninka, Bella Coola, Burmese, Cantonese, Carrier, Creek, English, French, German, Guaraní, Hualapai, Hungarian, Japanese, Kammu, Kinyarwanda, Korean, Koyukon, Lushai, Malay, Manchu, Mandarin, Marathi, Matses, Navajo, Olutec, Quechua, Shipibo‐Konibo, Sikuani, Svan, Tarascan, Tiddim Chin, Turkish, Yidiny, Yimas, and Yukaghir.

2. In the present case, the two are not *exactly* equivalent, since the present model uses a decay function (decay set to 0.5 for all simulations), simply for consistency with Ambridge et al. (2020). That said, a zero‐decay version of the model yields virtually identical results. For this reason, these results are not presented here, but the relevant plots can be downloaded from the OSF repository for the present study (https://osf.io/5sd2h/); specifically, the file BAL_Model Zero Decay.pdf from within the zip file Balinese_Modeling.zip (created using the R code V2 Balinese Original but Zero Decay.R, from within the same zip file).
